# Supplementary material for: Biogenesis of specialized lysosomes in differentiated keratinocytes relies on close apposition with the Golgi apparatus
Source: Cell Death Dis. 2024 Jul 11;15(7):496. doi: 10.1038/s41419-024-06710-w (PMC11239851; doi:10.1038/s41419-024-06710-w)

# Supplementary Information

# Biogenesis of specialized lysosomes in differentiated keratinocytes relies on close apposition with the Golgi apparatus

**Supplementary Figures:**

Figure S1.

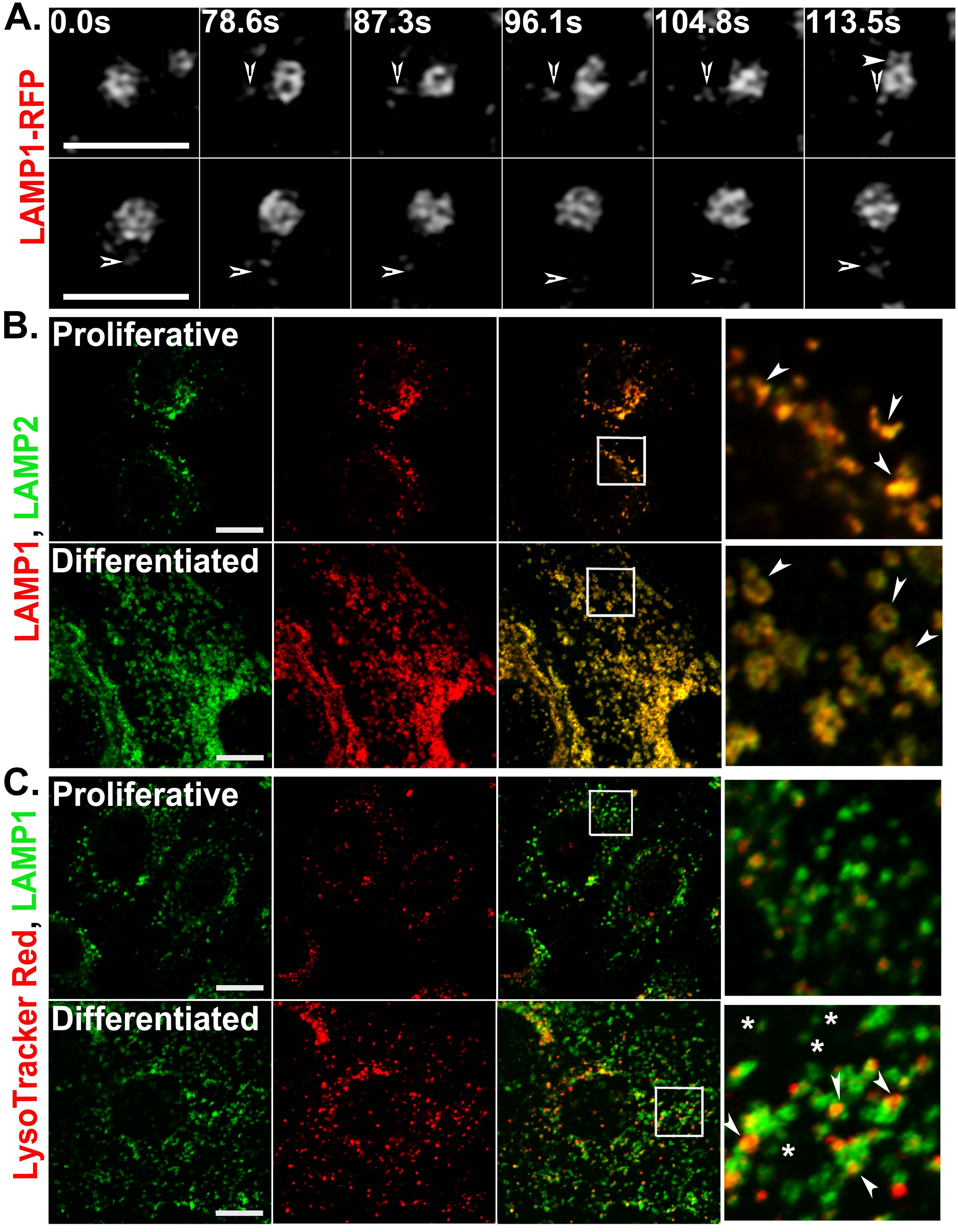
A.
B.
C.
0.0s
78.6s
87.3s
96.1s
104.8s
113.5s
LAMP1-RFP
LAMP1, LAMP2
Proliferative
Differentiated
LysoTracker Red, LAMP1
Proliferative
Differentiated

**Figure S1. Comparative analyses of the lysosomes of proliferative and differentiated keratinocytes.** (**A**) Time-lapse imaging of differentiated keratinocyte expressing LAMP1-RFP by Airyscan super-resolution microscope at 63X magnification. Arrowheads following the dynamics of small LAMP1 carriers. Two different events from the same cell are presented (top and bottom panel). Scale bar=2.5 μm. (**B-C**) IFM analysis of proliferative and differentiated keratinocytes co-immunostained for LAMP1 with either LAMP2 (B) or LysoTracker Red (C). Cells were imaged by Airyscan super-resolution microscope at 63X magnification. Insets represent 2.5 times magnified white boxed regions. Arrowheads point to the colocalization between the markers. White stars represent the LAMP1 structures that are negative for LysoTracker Red. Scale bar=10 μm.

# Figure S2.

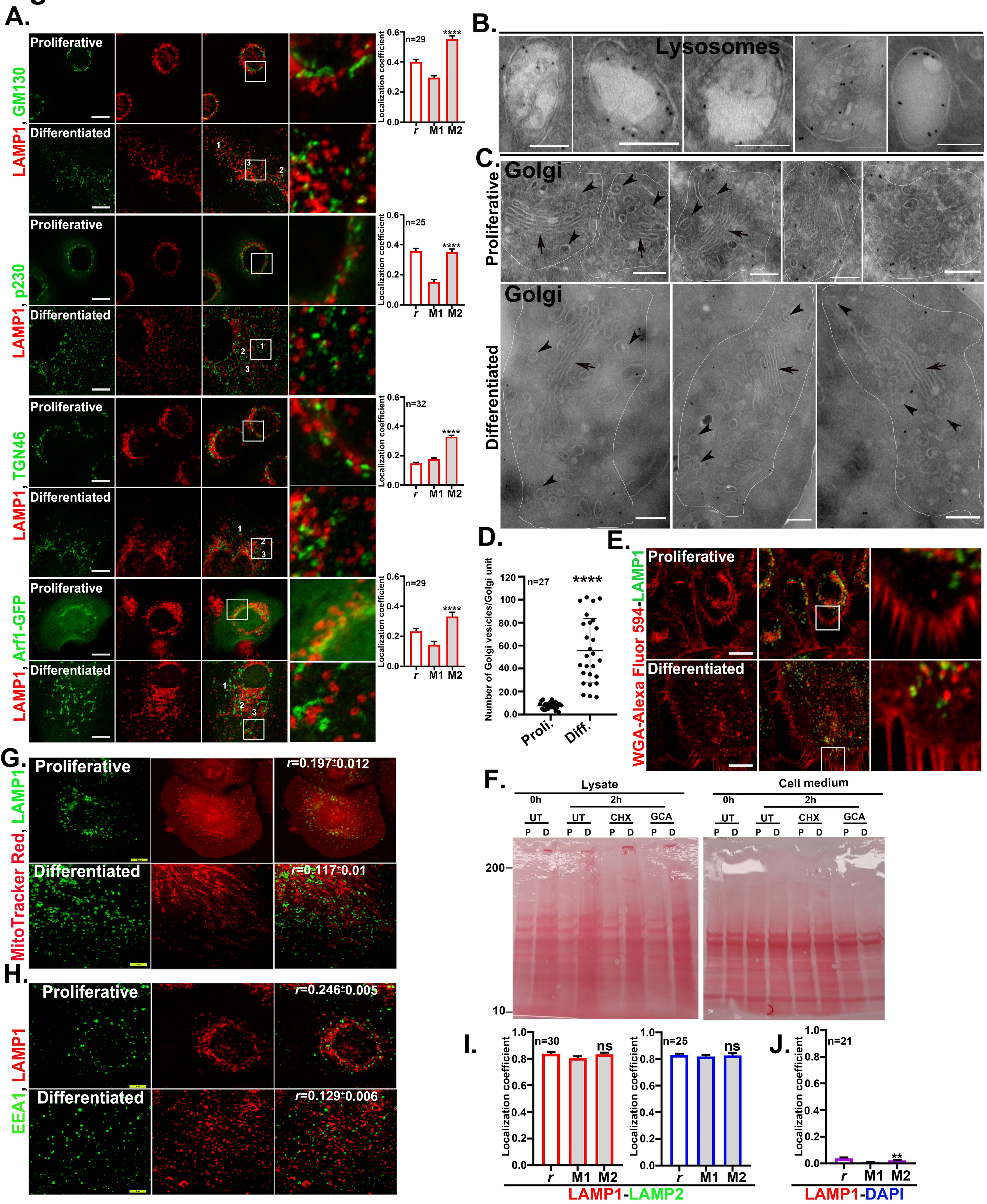

A.
B.
C.
D.
E.
F.
G.
H.
I.
J.
LAMP1, GM130
LAMP1, p230
LAMP1, TGN46
LAMP1, Arf1-GFP
Proliferative
Differentiated
Localization coefficient
n=29
n=25
n=32
n=29
r
M1
M2
****
Lysosomes
Golgi
Number of Golgi vesicles/Golgi unit
n=27
Proli.
Diff.
WGA-Alexa Fluor 594-LAMP1
Lysate
Cell medium
0h
2h
UT
CHX
GCA
P
D
200
10
MitoTracker Red, LAMP1
r=0.197±0.012
r=0.117±0.01
EEA1, LAMP1
r=0.246±0.005
r=0.129±0.006
n=30
n=25
n=21
ns
**
LAMP1-LAMP2
LAMP1-DAPI

**Figure S2. Presence of Golgi lysosome apposition in differentiated keratinocytes.** (**A**) IFM analysis of proliferative and differentiated keratinocytes co-stained for LAMP1 with either GM130, p230, TGN46 or expressed ARF1-GFP. Cells were visualized by Airyscan super-resolution microscope. The numbers (1-3) on the differentiated keratinocytes represent the magnified regions that are presented in Fig. 2B. Insets represent 2.5 times magnified white boxed regions. Pearson's correlation coefficient (*r*) and Manders' overlap coefficients (M1 and M2) in proliferative cells were calculated and plotted as mean±s.e.m. **** on the graphs indicates significant difference between M1 and M2 values. n=number of cells used for quantification. Scale bar=10 µm. (**B**) LAMP1-immunogold labelling of ultrathin cryosections to define lysosomes. Represented lysosomal structures are present in both proliferative and differentiated keratinocytes. Scale bar=200 nm. (**C**) Comparative Golgi morphology in proliferative and differentiated keratinocytes. Scale bar=200 nm. Black arrows pointing to the Golgi stacks, while arrowheads pointing the Golgi vesicles (GVs). Please note that the LAMP1 immunogold labelled sections were also used to analyze the Golgi morphology. Negative staining of the Golgi apparatus by LAMP1 confirms the specificity of LAMP1 immunolabelling in S2B. (**D**) Quantification of the number of associated GVs /unit of Golgi in proliferative and differentiated keratinocytes. n=27 Golgi from multiple experiments were quantified. The distribution of the values is shown; error bars: SD. **(E)** Proliferative and differentiated keratinocytes were labeled for WGA-Alexa flour 594, co-labelled for LAMP1. Cells were visualized by Airyscan super-resolution microscope at 63X magnification. Insets represent 2.5 times magnified white boxed region. Scale bar=10 µm. (**F**) Loading controls for the SUnSET assay presented in Fig. 2C. The total synthesis and secretion were measured with or without the presence of small molecules. P= proliferative and D = differentiated conditions. UT= untreated, CHX= cycloheximide, GCA= Golgicide-A. Total synthesis and secretion is shown by the lysate and in cell medium/supernatant respectively. (**G**-**H**) Proliferative and differentiated keratinocytes were co-immuno stained for LAMP1 with (G) MitoTracker Red or, (H) EEA1. Cells were visualized by Airyscan super-resolution microscope at 63X magnification. Scale bar=10 µm. (**I-J**) Proliferative and differentiated keratinocytes were co-immuno stained for LAMP1 with either (I) LAMP2 (red=proliferative; blue=differentiated) or (J) DAPI respectively. The localization coefficients (Pearson's correlation coefficient (*r*) and Manders' overlap coefficients, (M1 and M2) values were plotted as mean±s.e.m. n=the number of cells quantified from at least three independent experiments.

## Figure S3

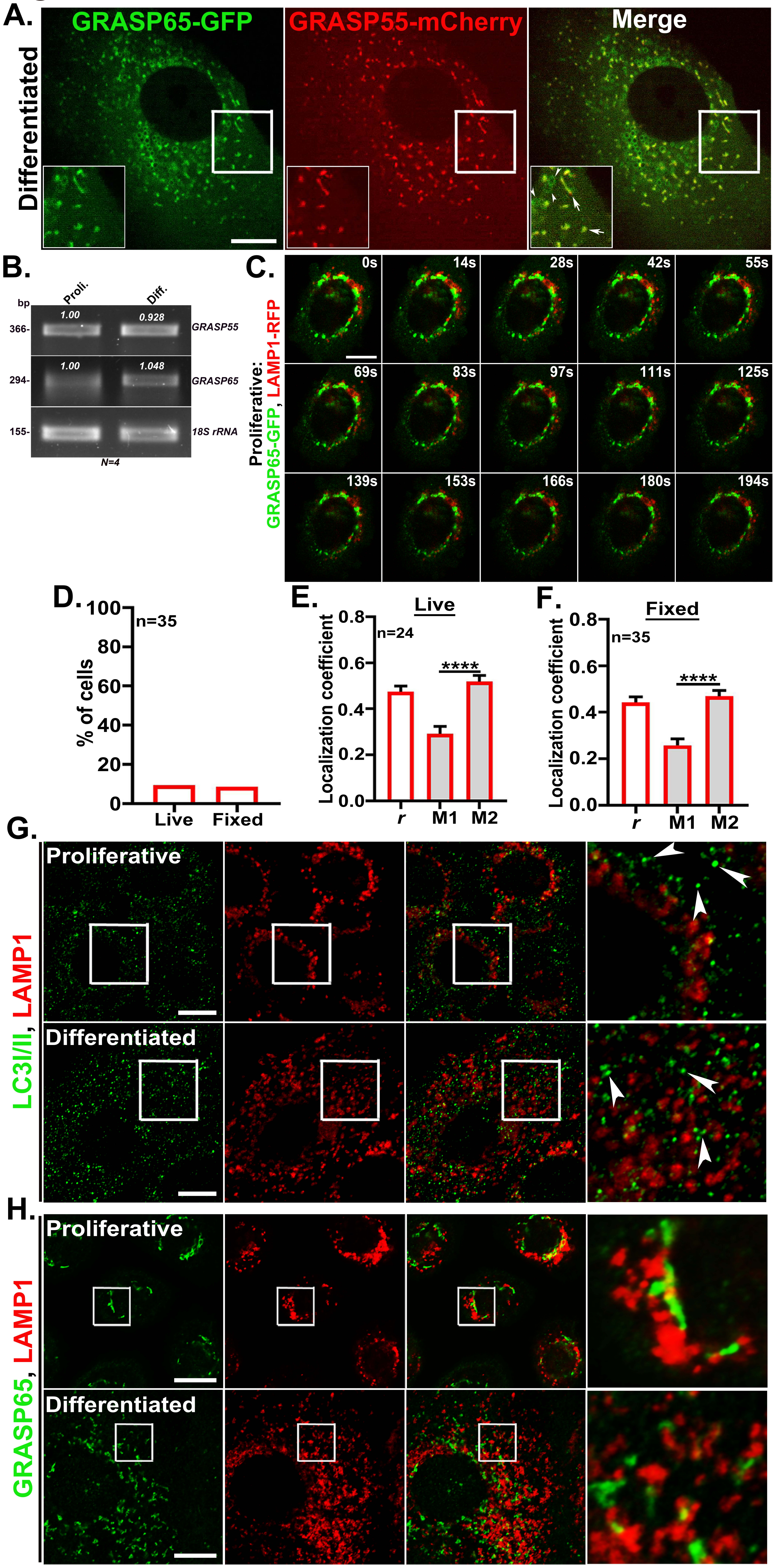

A.
Differentiated
GRASP65-GFP
GRASP55-mCherry
Merge
B.
Proli.
Diff.
bp
366-
294-
155-
1.00
0.928
1.048
GRASP55
GRASP65
18S rRNA
N=4
C.
Proliferative: GRASP65-GFP, LAMP1-RFP
0s
14s
28s
42s
55s
69s
83s
97s
111s
125s
139s
153s
166s
180s
194s
D.
% of cells
n=35
Live
Fixed
E.
Live
Localization coefficient
n=24
****
r
M1
M2
F.
Fixed
Localization coefficient
n=35
****
r
M1
M2
G.
LC3I/II, LAMP1
Proliferative
Differentiated
H.
GRASP65, LAMP1
Proliferative
Differentiated

**Figure S3. GRASP65 surrounds the lysosomes in differentiated keratinocytes.** (**A**) Differentiated keratinocytes expressing GRASP65-GFP and GRASP55-mCherry were imaged by a spinning disk microscope. Arrows and arrowheads represent respectively to the colocalization of GRASP65 with GRASP55 in dissociated Golgi stacks and, unique ring like structures of GRASP65 that are negative for GRASP55. Insets represent 2.5 times magnified white boxed regions. Scale bar=10μm. (**B**) mRNA expression of GRASP55 and GRASP65 in proliferative and differentiated keratinocytes was measured by semi quantitative-RT PCR. (**C**) Live cell imaging of proliferative keratinocyte expressing GRASP65-GFP and LAMP1-RFP by Airyscan super-resolution microscope. Scale bar=10 μm. (**D**) The graph represents % of proliferative cells having at least one GRASP65-GFP ring in live or in fixed state. n=number of cells. (**E**-**F**) Quantification of coefficients (Pearson's correlation coefficient, *r* and Manders' overlap coefficients, M1 and M2) between GRASP65-GFP and LAMP1-RFP in the proliferative keratinocytes in live (E) and, in fixed (F) conditions. The values were plotted as mean±s.e.m. n=number of cells from three independent experiments. (**G**) IFM analysis of proliferative and differentiated keratinocytes immunostained with LAMP1 and LC3 (G) arrowheads represent to the LC3-positive autophagosomes. (**H**) Co-immunostaining of endogenous GRASP65 with LAMP1. Cells were imaged by Airyscan super-resolution microscope. Insets represent 2.5 times magnified white boxed regions. Scale bar=10 μm.

Figure S4.

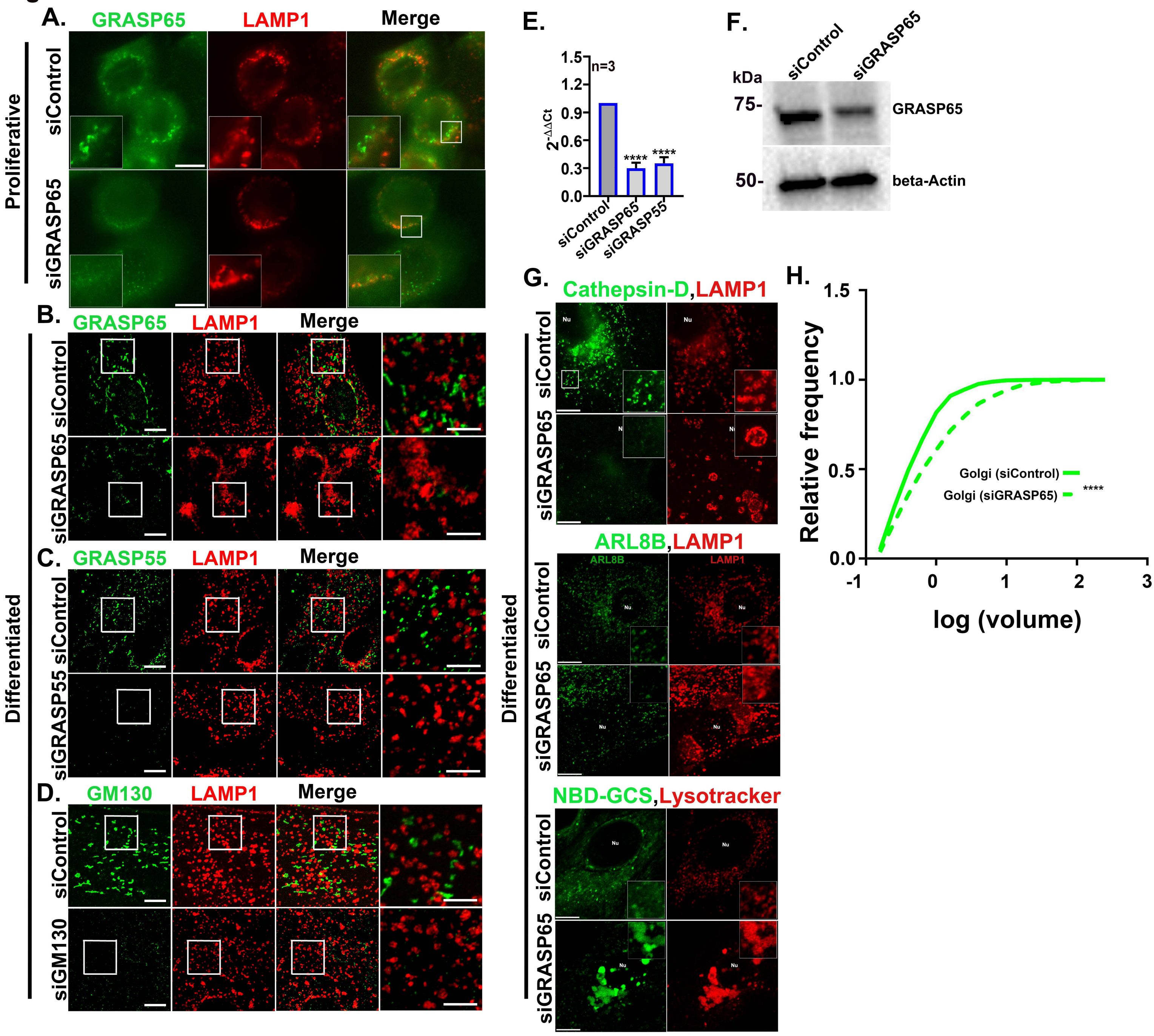

A.
GRASP65
LAMP1
Merge
Proliferative
siControl
siGRASP65
B.
GRASP65
LAMP1
Merge
Differentiated
siControl
siGRASP65
C.
GRASP55
LAMP1
Merge
siControl
siGRASP55
D.
GM130
LAMP1
Merge
siControl
siGM130
E.
n=3
$2^{-\Delta\Delta Ct}$
1.5
1.2
0.9
0.6
0.3
0.0
****
****
siControl
siGRASP65
siGRASP55
F.
siControl
siGRASP65
kDa
75-
50-
GRASP65
beta-Actin
G.
Cathepsin-D,LAMP1
siControl
siGRASP65
Nu
ARL8B,LAMP1
ARL8B
LAMP1
Differentiated
NBD-GCS,Lysotracker
H.
Relative frequency
1.5
1.0
0.5
0.0
-1
0
1
2
3
log (volume)
Golgi (siControl)
Golgi (siGRASP65)
****

**Figure S4. GRASP65 mediates Golgi-lysosome contacts in differentiated keratinocytes.** (**A**) IFM analysis of the control siRNA/GRASP65 siRNA treated proliferative keratinocytes using GRASP65 and LAMP1 co-immunostaining. (**B-D**) IFM analysis of the respective siRNA treated differentiated keratinocytes (as labelled). Cells were co immunostained for LAMP1 with either GRASP65 (B), or GRASP55 (C), or GM130 (D) and imaged by Airyscan super resolution microscope. Scale bar=10 μm. Insets represent 2.5 times magnified white boxed regions. Scale bar=5 μm. (**E**) GRASP65 and GRASP55 transcript analysis in respective siRNA treated condition, plotted as fold change. Values of 3 independent experiments were included. (**F**) Immunoblotting analysis of control siRNA and GRASP65 siRNA treated cells. Blots were probed with GRAPS65 or beta-actin (as internal control). (**G)** Characterization of the giant LAMP1-positive structures in GRASP65siRNA treated differentiated keratinocytes. Cells were stained for LAMP1 and Cathepsin-D, or Arl8B ($1^{st}$ and $2^{nd}$ panels). In the $3^{rd}$ panel, cells were incubated with NBD-glucosylceramide (GC) and lysotracker red. Scale bar=10 μm. The merged insets are presented in Fig. 4E. **(H)** Volumetric distribution analysis was performed as described in materials and methods. The log10 values were plotted as cumulative fractions. Statistical significance of the volume of the Golgi in control siRNA and GRASP65 siRNA treated cells were calculated by Mann-Whitney non parametric test.

Figure S5.

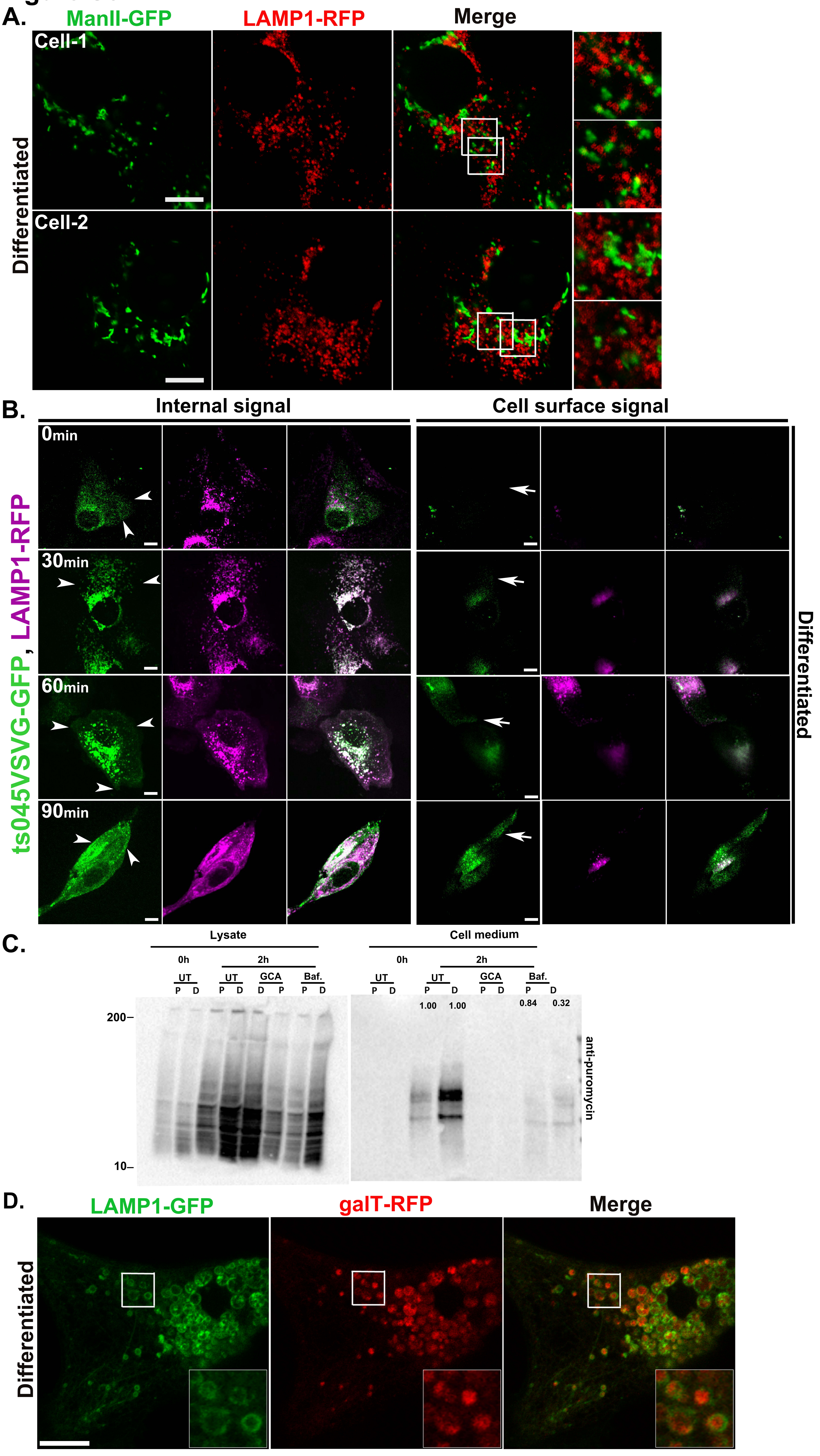
A.
ManII-GFP
LAMP1-RFP
Merge
Cell-1
Cell-2
Differentiated
B.
Internal signal
Cell surface signal
0min
30min
60min
90min
ts045VSVG-GFP, LAMP1-RFP
Differentiated
C.
Lysate
Cell medium
0h
2h
UT
GCA
Baf.
P D
1.00
1.00
0.84
0.32
200–
10–
anti-puromycin
D.
LAMP1-GFP
galT-RFP
Merge
Differentiated

**Figure S5. Differentiated keratinocytes produce specialized secretory lysosomes** (**A**) IFM analysis of differentiated keratinocytes expressing ManII-GFP and LAMP1-RFP. Cells were imaged by Airyscan super-resolution microscope. Insets representing two different regions (white boxes) were magnified 2.5 times and shown separately. Scale bar=10 µm. (**B**) IFM analysis of the co-expressed ts045VSVG-GFP and LAMP1-RFP. Post 20 hours of transfection, cells were shifted to a $40^0$C incubator with 5% CO2 and incubated overnight. The trafficking of VSVG then tracked at $32^0$C over the time points of 30 min, 60 min and 90 min. At the end of each time point cells were immediately fixed with 4% PFA. Cells were imaged by a Spinning disk microscope at 63X magnification. Scale bar=10 µm. (**C**) Total protein secretion from the proliferative and differentiated keratinocytes was measured by the SUnSET assay in presence or absence of the lysosomal inhibitor bafilomycin-A1. The total secretion was measured at 0h and 2h. P= proliferative and D = differentiated conditions. UT= untreated, GCA= Golgicide-A., Baf. =bafilomycin-A1. Total synthesis and secretion are shown by the signal in lysate blot and in cell medium/supernatant respectively. (**D**) IFM analysis of differentiated keratinocytes expressing LAMP1-GFP and galT-RFP in presence of bafilomycin-A1(50nM). Imaging was performed by Airyscan microscope at 63X magnification. Scale bar=10 µm.

Figure S6.

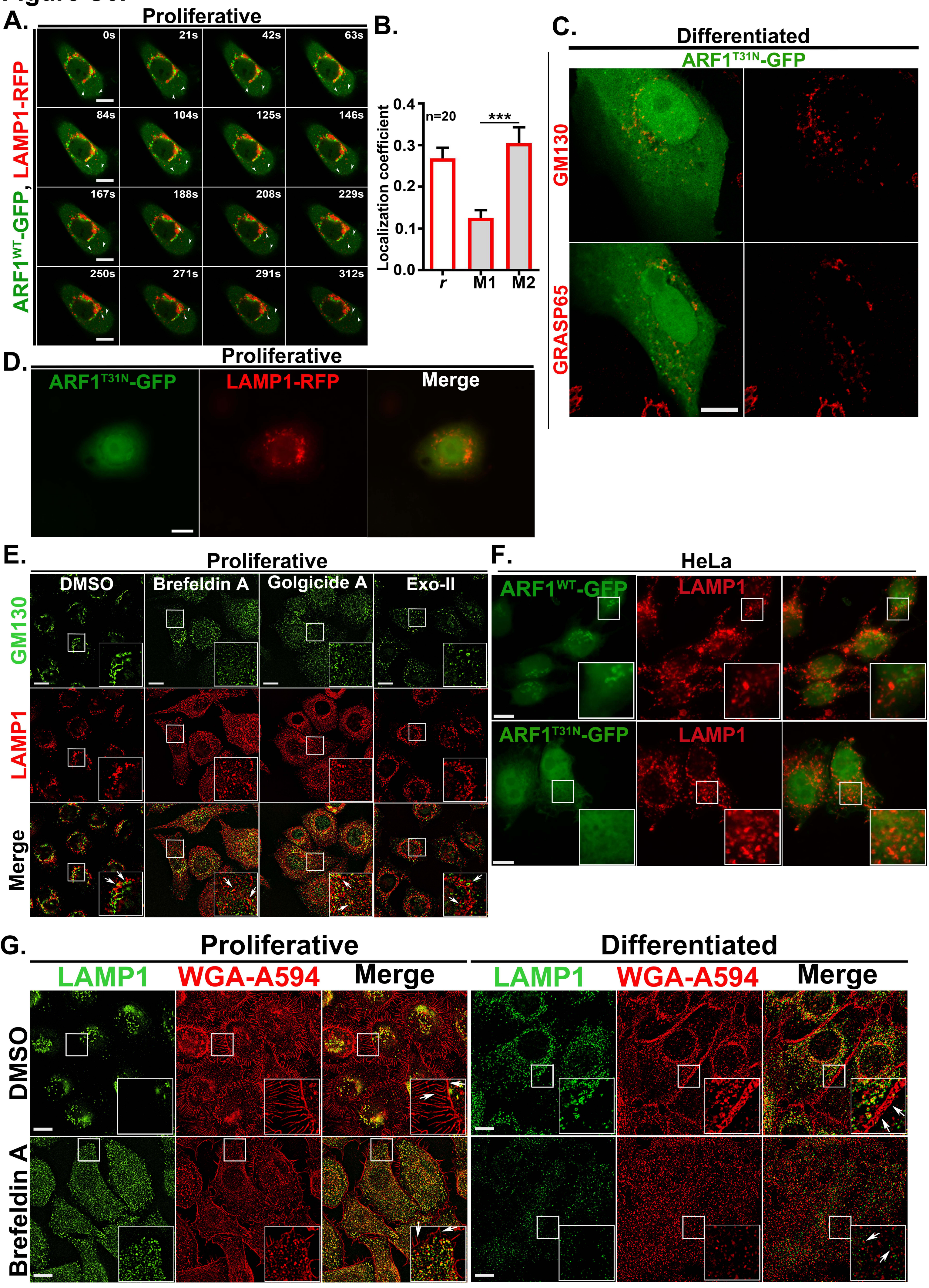
A.
Proliferative
ARF1WT-GFP, LAMP1-RFP
0s
21s
42s
63s
84s
104s
125s
146s
167s
188s
208s
229s
250s
271s
291s
312s
B.
Localization coefficient
n=20
***
0.0
0.1
0.2
0.3
0.4
r
M1
M2
C.
Differentiated
ARF1T31N-GFP
GM130
GRASP65
D.
Proliferative
ARF1T31N-GFP
LAMP1-RFP
Merge
E.
Proliferative
DMSO
Brefeldin A
Golgicide A
Exo-II
GM130
LAMP1
Merge
F.
HeLa
ARF1WT-GFP
LAMP1
ARF1T31N-GFP
LAMP1
G.
Proliferative
Differentiated
LAMP1
WGA-A594
Merge
LAMP1
WGA-A594
Merge
DMSO
Brefeldin A

**Figure S6. A functional Golgi maintains keratinocyte lysosomes.** (**A**) Time lapse analysis of proliferative keratinocytes expressing ARF1$^{WT}$-GFP and LAMP1-RFP. Live cell imaging was performed by Airyscan super-resolution microscope at 63X magnification. Scale bar=10µm. (**B**) localization coefficients (Pearson's correlation coefficient, *r* and Manders' overlap coefficients, M1 and M2) in proliferative cells between ARF1-GFP and LAMP1-RFP were plotted as mean±s.e.m. n= the number of cells quantified from at least three independent experiment. (**C**) IFM analysis of Golgi morphology of differentiated keratinocytes expressing ARF1$^{T31N}$-GFP, immuno stained either for GM130 or GRASP65. Scale bar=10 µm. (**D**) analysis of lysosome morphology of proliferative keratinocyte co-expressed with ARF1$^{T31N}$-GFP and LAMP1-RFP. Scale bar=10 µm. (**E**) Proliferative keratinocytes were treated with pharmacological inhibitors as described in materials and methods. Cells were fixed and stained for GM130 and LAMP1. Insets represent 2.5 times magnified white boxed regions. Arrows indicate to the Golgi and lysosome morphology in all treatment conditions. Scale bar=10 µm. (**F**) HeLa cells were transfected with either ARF1-GFP or ARF1$^{T31N}$-GFP and immunostained with LAMP1. Scale bar=10 µm. (**G**) Proliferative and differentiated keratinocytes either treated with DMSO or BFA, fixed and stained for WGA Alexa flour 594 (WGA-A594) and LAMP1. Insets represent 2.5 times magnified white boxed regions. Arrows indicate to the effect of BFA on the plasma membrane binding of WGA. In all images: scale bar=10 µm.

**Supplementary Videos:**
**Video S1:** Live cell imaging of differentiated keratinocyte expressing LAMP1-RFP. The scale bar and time frames are mentioned in the video.

**Video S2:** Live cell imaging of differentiated keratinocytes expressing GRASP65-GFP and LAMP1-RFP. The scale bar and time frames are mentioned in the video.

**Video S3:** Live cell imaging of proliferative keratinocyte expressing GRASP65-GFP and LAMP1-RFP. The scale bar and time frames are mentioned in the video.

**Video S4:** Live cell imaging of differentiated keratinocyte expressing ARF1-GFP and LAMP1-RFP. The scale bar and time frames are mentioned in the video.

**Video S5:** Live cell imaging of proliferative keratinocyte expressing ARF1-GFP and LAMP1-RFP expressed. The scale bar and time frames are mentioned in the video.

**Supplementary Immunoblots:** The full-length uncropped version of immunoblots.

## Figure 2C: protein secretion assay

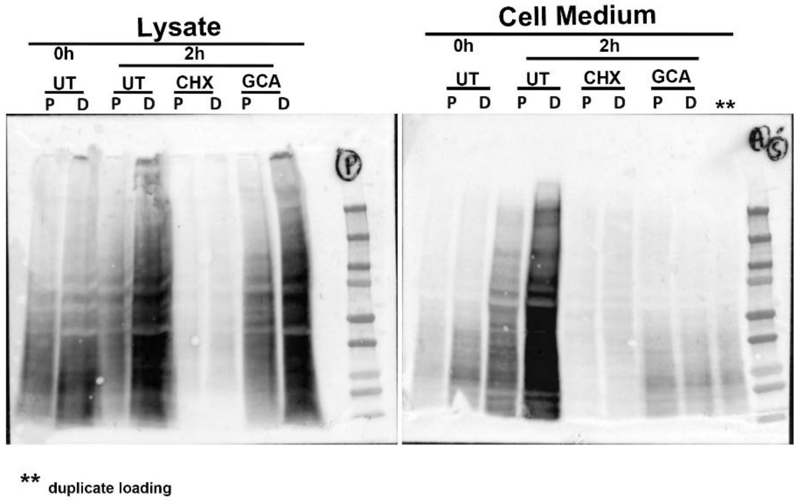


## Figure S5C: protein secretion assay

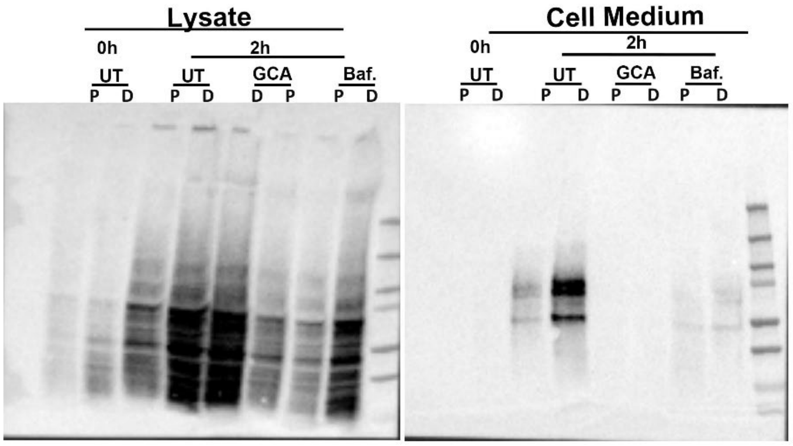


## Figure S4F

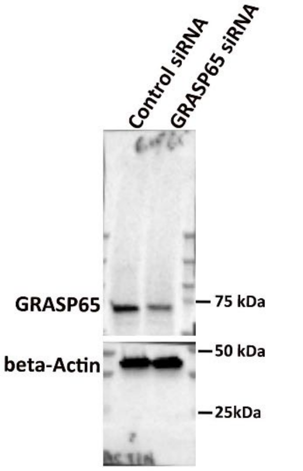

Supplement: Supplementary file 1 — Supplementary Information [file 41419_2024_6710_MOESM1_ESM.pdf]
